# Comparison of multiple modalities for drug response prediction with learning curves using neural networks and XGBoost

**DOI:** 10.1093/bioadv/vbad190

**Published:** 2023-12-23

**Authors:** Nikhil Branson, Pedro R Cutillas, Conrad Bessant

**Affiliations:** School of Biological and Behavioural Sciences, Queen Mary University of London, London E1 4NS, United Kingdom; Digital Environment Research Institute, Queen Mary University of London, London E1 1HH, United Kingdom; Centre for Genomics and Computational Biology, Barts Cancer Institute, Queen Mary University of London, London EC1M 6BQ, United Kingdom; School of Biological and Behavioural Sciences, Queen Mary University of London, London E1 4NS, United Kingdom; Digital Environment Research Institute, Queen Mary University of London, London E1 1HH, United Kingdom

## Abstract

**Motivation:**

Anti-cancer drug response prediction is a central problem within stratified medicine. Transcriptomic profiles of cancer cell lines are typically used for drug response prediction, but we hypothesize that proteomics or phosphoproteomics might be more suitable as they give a more direct insight into cellular processes. However, there has not yet been a systematic comparison between all three of these datatypes using consistent evaluation criteria.

**Results:**

Due to the limited number of cell lines with phosphoproteomics profiles we use learning curves, a plot of predictive performance as a function of dataset size, to compare the current performance and predict the future performance of the three omics datasets with more data. We use neural networks and XGBoost and compare them against a simple rule-based benchmark. We show that phosphoproteomics slightly outperforms RNA-seq and proteomics using the 38 cell lines with profiles of all three omics data types. Furthermore, using the 877 cell lines with proteomics and RNA-seq profiles, we show that RNA-seq slightly outperforms proteomics. With the learning curves we predict that the mean squared error using the phosphoproteomics dataset would decrease by ∼15% if a dataset of the same size as the proteomics/transcriptomics was collected. For the cell lines with proteomics and RNA-seq profiles the learning curves reveal that for smaller dataset sizes neural networks outperform XGBoost and *vice versa* for larger datasets. Furthermore, the trajectory of the XGBoost curve suggests that it will improve faster than the neural networks as more data are collected.

**Availability and implementation:**

See https://github.com/Nik-BB/Learning-curves-for-DRP for the code used.

## 1 Introduction

Stratified medicine primarily aims to improve treatment outcomes for patients and decrease treatment costs. This is done by tailoring treatments to certain subgroups, or individuals, based on biomarkers associated with disease mechanisms.

In the search for a stratified approach to cancer treatment, studies of cancer cell lines have become crucial because of the lack of publicly available clinical datasets with large enough sample sizes (Sharifi-Noghabi *et al.* 2021). This is despite cell lines lacking many of the biological complexities of clinical samples. Therefore, large-scale studies have been undertaken to profile numerous cell lines and screen each of their responses when treated with different drugs (Barretina *et al.* 2012, Yang *et al.* 2012, Basu *et al.* 2013, Seashore-Ludlow *et al.* 2015). These studies include known and possible anti-cancer drugs and the data from them have been made publicly available.

### 1.1 Drug response prediction

In the context of cancer cell lines, drug response prediction (DRP) is the process of predicting how effective a given drug is at killing a given cell line. This is done by predicting efficacy metrics that capture how effective a given drug is, typically as reported by one of the large-scale studies (Barretina *et al.* 2012, Yang *et al.* 2012, Basu *et al.* 2013, Seashore-Ludlow *et al.* 2015).

There have been many studies that have used the genomic or transcriptomic profiles of cancer cell lines, combined with drug profiles, for DRP using machine learning (Baptista *et al.* 2021, Chen and Zhang 2021, Partin *et al.* 2023). This is despite an expectation that proteomics and phosphoproteomics data should be more suitable because drugs target proteins, not DNA or RNA (Poulos *et al.* 2022). A 2021 review paper (Baptista *et al.* 2021) focussed on 15 DRP studies: only one used proteomics, and none used phosphoproteomics. Similarly, another 2021 review paper (Chen and Zhang 2021) discussed 17 studies, of which two used proteomics data, and none used phosphoproteomics. A third review paper (Partin *et al.* 2023) focussed on deep learning (DL) and looked at 60 studies. It found just one study used proteomics, and again none used phosphoproteomics. This is partly because there are few datasets with proteomics and phosphoproteomics cancer cell line profiles. However, recently a proteomics and a phosphoproteomics dataset, with the appropriate number of cell lines for DRP, have been made publicly available (Gerdes *et al.* 2021, Gonçalves *et al.* 2022), so the potential for these modalities to improve DRP has not yet been fully explored. At 949 different cell lines profiled, the size of the proteomics dataset is similar to the transcriptomic datasets that have previously been used for DRP. However, the size of the phosphoproteomics dataset is more limited, sitting at 48 different cell lines.

### 1.2 Learning curves

In most areas of medical research, including DRP, dataset size is a major challenge because data collection is typically both time-consuming and expensive. In multiple domains of machine learning, it has been shown that as dataset size increases model performance improves (Hestness *et al.* 2017, Viering and Loog 2022). Thus, it is a general rule of machine learning that large amounts of data are needed for state-of-the-art performance. However, how much of an improvement collecting more data is expected to make depends on the algorithm used and the specific application. Therefore, for each algorithm and application, there is a different trade-off between model performance and data collection costs. Learning curves can be used to estimate if and how the performance of a model would increase with more data.

An individual learning curve is defined as a plot of the performance of a given learning algorithm for different values of training set sizes, *n*. The model performance of interest is its performance on unseen data, known as the generalization performance. Thus, the performance is calculated for multiple values of *n* to produce an individual learning curve. However, in practice, multiple training sets, $T_{n}$, of size *n* are typically used to create many individual learning curves that are then averaged over (Ng and Jordan 2001, Mukherjee *et al.* 2003, Kim 2009, Partin *et al.* 2021). This is because there can be large deviations between different sets of $T_{n}$. For simplicity, the remainder of this report will refer to the averaged individual learning curves as just the learning curve.

Learning curves can be used as an indication of how the generalization error will change with increased dataset size. This allows for a more informed cost-benefit analysis when deciding if more data should be collected for a given problem. This is particularly useful for problems where data collection is challenging, such as DRP. Previous work using learning curves for a similar purpose includes estimating how additional data would improve the generalization error for a decision tree across multiple datasets in the UCI Machine-Learning Repository (Frey and Fisher 1999) and tumour classification using transcriptomics and support vector machines (Mukherjee *et al.* 2003).

Learning curves have also been used to estimate how the performance for DRP using machine learning would improve with the collection of more data (Partin *et al.* 2021). However, this has only been done for RNA-seq data using mixed-set testing. In mixed-set testing drug cell line pairs are randomly split into training and testing sets. Therefore, applications of these models are suitable for drug repurposing, because they are trained on every drug and cell line in the dataset and tested on unseen combinations of these drugs and cell lines.

In this article, we compare phosphoproteomics, proteomics, and RNA-seq data for DRP using cancer-blind testing. In cancer-blind testing, models are evaluated on cell lines that are not in the training set. Furthermore, in addition to transcriptomics, we create and compare learning curves for both phosphoproteomics and proteomics. It is important to note that cancer-blind testing has not previously been evaluated using learning curves.

A model that performs well using cancer-blind testing could be used to find candidate drugs that might be effective for unseen cell lines. Cancer-blind testing also indicates how good a model would be in a stratified medicine context. This is because in, clinical use, the model would be used to predict the responses of a patient that the model had not been trained on.

We conduct our analysis using both neural networks (NNs) and the gradient-boosting algorithm, XGBoost. This is because multiple studies have shown that NNs outperform other machine-learning algorithms for DRP (Chang *et al.* 2018, Sakellaropoulos *et al.* 2019, Liu *et al.* 2020, 2022, Zhu *et al.* 2021). However, this success might be because drugs are represented most faithfully by unstructured data and NNs have proven extremely successful in domains with unstructured data, such as images, text, and audio (He *et al.* 2016, Devlin *et al.* 2019, Purwins *et al.* 2019, Liu *et al.* 2021). However, the input omics data are tabular data, each sample has the same set of features, and for tabular data gradient-boosted models have shown superior performance to DL (Borisov *et al.* 2022, Shwartz-Ziv and Armon 2022). Therefore, it is unclear whether DL is best suited for omics data. Furthermore, XGBoost has rarely been used for DRP (Partin *et al.* 2023). Therefore, NNs and XGBoost are used here and are compared against a simple benchmark.

## 2 Methods

### 2.1 Datasets

We used three different types of input omics data to represent the cell lines:

1. RNA-seq data from the Genomics of drug sensitivity in cancer database (Yang *et al.* 2012). This dataset contains 17,417 gene expression values for 970 cell lines.
2. Proteomics data from Gonçalves *et al.* (2022). This dataset contains the relative abundance of 8,457 proteins for 949 cell lines.
3. Phosphoproteomics data from Gerdes *et al.* (2021). This dataset contains abundance measurements of 22,804 phosphopeptides for 48 cell lines.

Both the downloaded RNA-seq and proteomics data were already preprocessed such that the data were normalized and log2 scaled. The downloaded phosphoproteomics data were normalized by cell line, using standard scaling as was done in Gerdes *et al.* (2021). The data were also log2 scaled. These steps were taken to correct for batch effects. There were only missing values in the downloaded proteomics data, which contained 38% missing values. These missing values were replaced with zeros, as was previously done for DRP using proteomics (Gonçalves *et al.* 2022). This is because these missing values are assumed to indicate that the protein abundance is below the limit of quantitation.

To reduce the dimensionality of the omics data, we used the 1,000 landmark genes identified in Subramanian *et al.* (2017), as it has been shown that these landmark genes could recover the vast majority of the transcriptome (Subramanian *et al.* 2017). Furthermore, they have been successfully used for DRP (Partin *et al.* 2021). We used a similar feature selection process for each omics dataset.

For features in the RNA-seq dataset, we used the overlap of the 908 landmark genes and the genes in the RNA-seq dataset. For the proteomics dataset, we used the overlap of the 721 proteins that were coded for by the landmark genes and the proteins in the proteomics dataset. From the phosphorylation dataset, we used the overlap of the 1,064 phosphopeptides that were on a protein coded for by one of the landmark genes and are a target of one of the landmark genes [as defined by Omnipath (Türei *et al.* 2016)] and the phosphopeptides in the phosphoproteomics dataset. We verified that this feature selection method did not bias one omics type over another by using three other feature selection methods as described in Supplementary Table S1.

For drug response/target data, IC50 values from the GDSC1 database (Yang *et al.* 2012) were used. These IC50 values have been natural logarithm transformed. Thus, the ln(IC50) values will be used in this report to measure drug effectiveness and in the remainder of this article IC50 will be used to refer to ln(IC50) values for simplicity. Any cell lines in the omics datasets without any target values were removed from all datasets. To represent the drugs a simple column vector, with no information about the drugs’ molecular properties, was created via one-hot encoding. This was to distil the comparison between the omics data.

The phosphoproteomics dataset covered significantly fewer cell lines than the other datasets. Therefore, we considered two different cell line sets (CLSs) in the subsequent work. Firstly, the drug cell line pairs for the intersection of the 877 cell lines in the RNA-seq and proteomics dataset, $CLS_{877}$. Secondly, the drug cell line pairs for the intersection of the 38 cell lines in all three omics datasets, $CLS_{38}$.

### 2.2 Drug response prediction

To make the definition of DRP more concrete, consider a training dataset of size *n*, $T_{n}={\left\{x_{c,i},x_{d,i},y_{i}\right\}}_{i=1}^{n}$. Here $x_{c,i}$ is a $d_{1}$-dimensional vector, and $x_{d,i}$ is a $d_{2}$-dimensional vector such that $x_{c,i}\in R^{d_{1}}$ and $x_{d,i}\in R^{d_{2}}$. Furthermore, $y_{i}$ is the IC50 value associated with the $i^{th}$ cell line drug pair thus, $y_{i}\in R$. Here, $x_{c,i}$ is the representation of the $i^{th}$ cell line for a specific data type. For example, for the proteomics data, $x_{c,i}$ is the protein abundance for the proteins that were coded for by the landmark genes thus, $x_{c,i}\in R^{721}$. Furthermore, $x_{d,i}$ represents the $i^{th}$ one-hot encoded drug input. Thus, $x_{d,i}\in R^{345}$. A model, *M*, created by a learning algorithm takes $x_{c,i}$ and $x_{d,i}$ as inputs and predicts $\hat{y_{i}}$ for the corresponding IC50 value such that $M(x_{c,i},x_{d,i})=\hat{y_{i}}$.

A limitation of DRP comes from the fact that the omics profiles are measured once for a given cell line, rather than before that cell line is treated with a drug. Therefore, for a given omics type, there is only one omics profile for each cell line but multiple drug responses to predict. This means that the datasets used have multiple repeated input vectors. For example, the $i^{th}$ cell line, $x_{c,i}$ can have 345, different target values it could be used to predict. However, the pair $x_{c,i},x_{d,i}$ corresponds to the unique target value $y_{i}$.

### 2.3 Machine-learning algorithms

We used two machine-learning algorithms to create the models for DRP: a NN, implemented in TensorFlow Keras (Abadi *et al.* 2015, Chollet 2015) and a gradient-boosted decision tree implemented using the XGBoost library (Chen and Guestrin 2016a). We used the mean squared error (MSE) as the loss function for both algorithms. The NN architecture we created and used is shown in Supplementary Fig. S1. For both algorithms, we performed a random search of 15 trials to optimize the model’s hyperparameters and chose the model that performed the best on a held-out validation set (Bergstra and Bengio 2012). The hyperparameter search spaces are detailed in the Supplementary Material. We also used early stopping for both algorithms with the held-out validation set.

### 2.4 Drug average benchmark

We created a simple rule-based algorithm to be used as a benchmark. The benchmark did not use any omics data. Instead, predictions for IC50 values for a given drug in the evaluation set were simply calculated as the mean of all IC50 values for that drug in the training set. For the rare case, where a drug was in the evaluation set but not in the training set the predicted IC50 value was the mean of all IC50 values in the training set.

We hypothesized this benchmark was effective because there are drugs that are generally effective at killing most cell lines, and other drugs that are ineffective for most cell lines. Therefore, the mean behaviour of the drug on the training set should capture this pattern. More importantly, it allows us to see if adding omics data improve performance.

### 2.5 Learning curve regions

Supplementary Figure S2 is a sketch showing three regions that are typically seen in empirical learning curves. These regions were identified in Hestness *et al.* (2017) and are listed below.

1. The small data region. The model performance is poor, and often comparable to random guessing, due to insufficient dataset size.
2. The power-law region. The model performance follows a power-law equation where increasing the dataset size improves the performance.
3. The irreducible error/diminishing returns region. Additional data improves model performance at a slower rate than in the power-law region. Furthermore, the generalization error is expected to reach a lower bound that will be non-zero due to Bayes error and other factors. For example, incorrect IC50 values.

The power-law region is typically described by the equation

(1) $$\epsilon (n)=\alpha n^{\beta }.$$

Therefore, in a log–log plot of a learning curve, the power-law region will be a straight line, log(ϵ)=βlog(n) + log(α), as shown in Supplementary Fig. S2.

By exploiting the fact that the same learning regions are seen in multiple areas of machine learning, learning curves can be used to predict the extent to which more data might improve a model’s performance. This allows for the comparison of how a model’s performance might improve with more data. For example, consider two models, $m_{1}$ and $m_{2}$, and their associated learning curves. $LC_{1}$ and $LC_{2}$. Let the models both have the same performance for the maximum training dataset size available and be in the power-law region. Additionally, let the slope of the $LC_{1}$ be steeper than $LC_{2}$, for a log–log plot. The learning curves suggest that as the dataset size increases $m_{1}$ will start to outperform $m_{2}$. This is because the literature suggests the trajectory of these learning curves will continue on the same paths as the training set size is increased. A similar type of extrapolation was made in Partin *et al.* (2021). This example illustrates the power of learning curves compared to a single performance metric. However, it is important to note that the extrapolations are only predictions because there is no reliable way of knowing when the power-law region will end.

### 2.6 Learning curve generation

The method we used to create the learning curves is similar to the method used in Partin *et al.* (2021). Consider a dataset *D* with drug cell line pairs and associated IC50 values. *D* was split into three sets a: training (*T*), validation (*V*), and testing (*E*) set, with the key property that no cell line was in more than one set. To create a learning curve, *T* was split into *K* different sized subsets of size *n*, ${\left\{T_{n_{k}}\right\}}_{k=1}^{K}$. For a given learning algorithm, at each $T_{n_{k}}$ a hyperparameter optimized model was selected using *V*. The performance of the model was then measured on *E*. This gave the generalization error of the model for a given training set size, $s_{n_{k}}$. The above process was repeated 30 times, where *D* was first shuffled before splitting. The learning curve was found by taking the mean of $s_{n_{k}}$ across the repeats. The full details of the above method are given in the Supplementary Material.

### 2.7 Comparing all modalities using maximum training dataset sizes

We also performed an in-depth comparison of all three modalities using the maximum training dataset size, $T_{n_{K}}$. For this analysis, we took the performance results from the learning curves at the final training dataset size $T_{n_{K}}$. We then analysed the performance for each of the 30 different train test splits and the average performance across these splits. For $CLS_{877}\;T_{n_{K}}=198,\;668$ and $CLS_{38}\;T_{n_{K}}=6,949$.

## 3 Results and discussion

### 3.1 Comparisons of all modalities using maximum training dataset sizes

Table 1 shows the MSE on the test sets for XGBoost and NNs across all modalities. These models were trained using the full training sets. The table shows these results for both $CLS_{877}$ and $CLS_{38}$. The table also shows the average ranking for a given CLS and algorithm across the 30 train test splits used. Model errors were normally distributed for every dataset and model type (see Supplementary Figs S3 and S4).

**Table 1.** MSE and average ranking across the 30 test train split evaluated using the test sets and trained using the full training sets for $CLS_{877}$ and $CLS_{38}$.^a^

Cell line set	Data type	MSE XGBoost	Average ranking	MSE neural network	Average ranking
$CLS_{877}$	RNA-seq	**1.54±0.012**	1.2	1.61 ± 0.012	1.3
	Proteomics	1.56±0.012	1.8	1.64±0.014	1.7
					
	Phosphoproteomics	2.00±0.04	2.5	**1.84±0.05**	2.0
$CLS_{38}$	RNA-seq	1.93±0.05	1.8	1.86±0.05	1.8
	Proteomics	1.90±0.05	1.7	1.88±0.05	2.2

a The benchmark had MSE =1.88±0.02 for $CLS_{877}$ and 2.10±0.02 for $CLS_{38}$. Bold values give the best metrics for a given cell line set.

**Figure 1.**
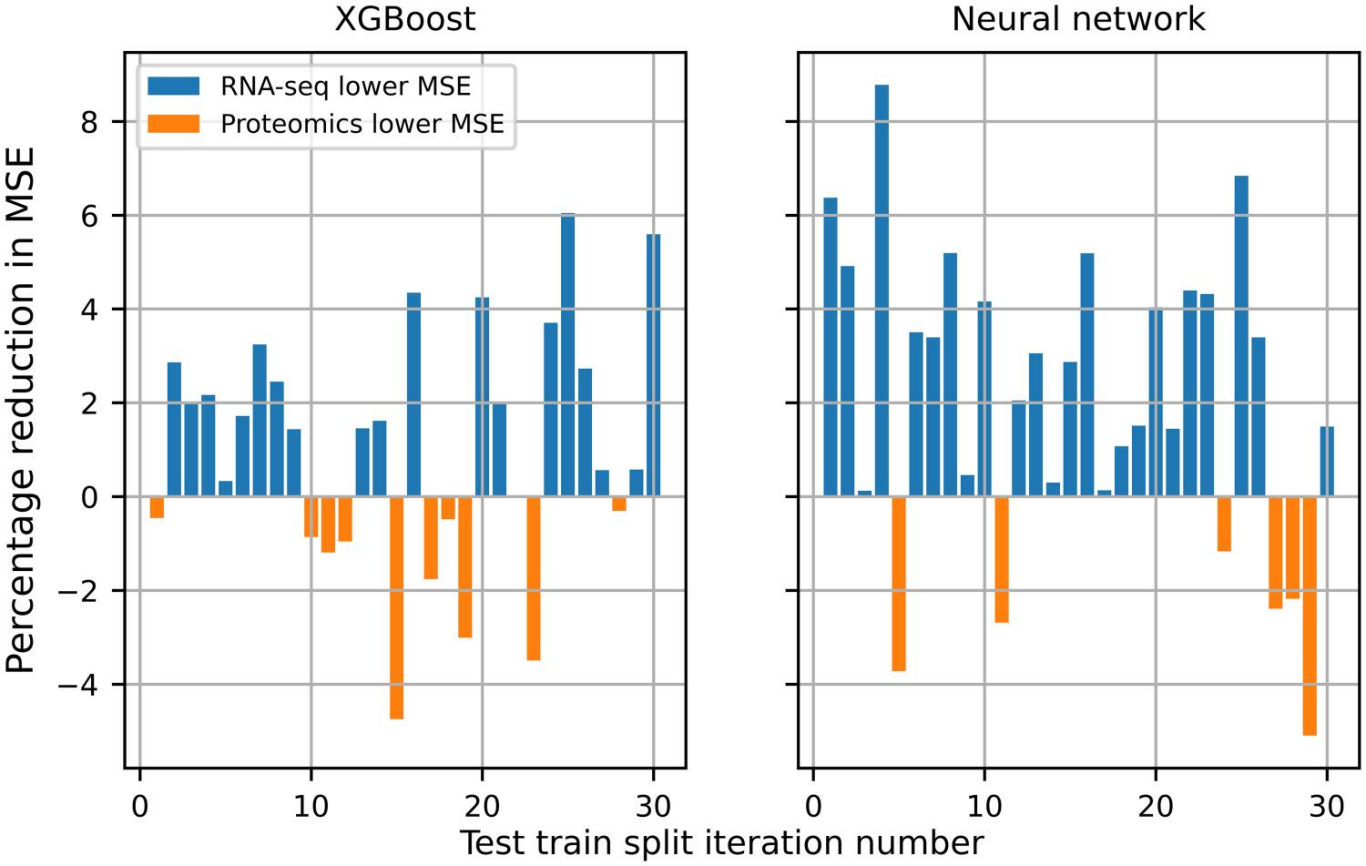
Figure to show the reduction in MSE, between RNA-seq and proteomics, on the 30 different test sets. The figure shows that RNA-seq outperforms proteomics for 25 and 20 of the test train splits for XGBoost and NNs, respectively.

For $CLS_{877}$, the benchmark had MSE =1.88 ± 0.02. Therefore, for $CLS_{877}$, all combinations of modalities and learning algorithms outperformed the benchmark by at least 13%. The table also shows that for both modalities, XGBoost had a ∼5% lower MSE than NNs. Furthermore, for a given algorithm RNA-seq slightly outperformed proteomics, as shown by the MSE and the average rankings. This agrees with previous results for DRP using proteomics (Gonçalves *et al.* 2022). Figure 1 shows the percentage reduction in MSE between RNA-seq and proteomics for the 30 different test train splits, for XGBoost and NNs. For example, for the $19^{th}$ test train split for XGBoost it shows that RNA-seq has ∼4% reduction in MSE compared with proteomics, on the testing set. Thus, Fig. 1 shows RNA-seq outperforms proteomics on 83% and 67% of the test train splits for XGBoost and NNs, respectively.

Table 1 also shows that the test train split has a large impact on performance. For example, the standard deviation in the MSE for both data types for XGBoost is 0.07. This variation, 5% of the MSE, is comparable in size to the difference in performance between the two data types for a given test train split, as shown by Fig. 1. Furthermore, the large variation in performance is reflected by the overlapping standard errors in Table 1 for XGBoost.

These results confirm our hypothesis that XGBoost might outperform NNs based on previous studies (Chen and Guestrin 2016b, Borisov *et al.* 2022, Shwartz-Ziv and Armon 2022).

For $CLS_{38}$, the benchmark had MSE =2.10±0.06. Therefore, for $CLS_{38}$ all combinations of modalities and learning algorithms outperformed the benchmark by between 5% and 12%. The table shows NNs with phosphoproteomics data had the lowest overall MSE. However, it performed very similarly to RNA-seq as shown by the lower average ranking of RNA-seq.

For $CLS_{38}$, the test train split has an even larger impact on performance than for $CLS_{877}$, as shown by the much larger standard errors. This is expected because the significantly smaller dataset size leads to bigger variations between different test train splits. Furthermore, because the models have fewer cell lines to learn from in this dataset any difference between the data types will be harder to learn than in $CLS_{877}$. This can be shown by separately considering the pairwise average rankings for the data types in $CLS_{38}$. Comparing just RNA-seq and proteomics for XGBoost the average ranking for both data types is 1.5. This is in contrast to 1.2 versus 1.8 for $CLS_{877}$. This shows how decreasing the data set size causes the performance difference to decrease. Similarly, for NNs and $CLS_{38}$ RNA-seq has an average ranking of 1.4 compared with 1.6 for proteomics. For $CLS_{877}$, the average rankings are 1.3 and 1.7, again showing the decrease in performance differences for the smaller dataset.

These results suggest that it is likely that phosphoproteomics will show a larger difference in performance compared with the other data types if a dataset of a similar size, 1,000 cell lines, were available. Furthermore, they suggest that the performance would be comparable to, or better than, those obtained for RNA-seq on such a dataset.

In the above analysis, the phosphorylation data included multiple sites per protein. We included multiple sites because, fundamentally, a phosphorylation measurement is a marker of kinase activity for the kinase(s) that phosphorylated that specific phosphorylation site and different kinases can phosphorylate different sites on the same protein. Furthermore, phosphosites at different locations on a protein may also affect the conformation or activity of that protein in different ways, so may have functional importance. To verify that keeping multiple sites on each protein did not mean we had redundant features we experimented using only one site per protein and found a large decrease in performance.

We further explored whether the cause of the strong performance of phosphoproteomics is due to it having slightly more features than RNA-seq by randomly subsampling the number of features down to 908 features, the same number of RNA-seq features used. However, we did not find this had an impact on performance. Instead, this suggests that the performance improvement is due to unique information in the phosphoproteomics data.

We also investigated model performance on a per-drug basis. We found that three of the 10 drugs whose response NNs struggled to predict the most, and 6 of the 10 drugs whose responses were predicted most accurately, were shared across all three omics types. These drugs are shown in Supplementary Fig. S5. We also found that the drugs whose response was more accurately predicted have lower variability in their IC50 values than those with poorly predicted responses. This is shown by Supplementary Figs S5 and S6. Thus, the models perform better for drugs with lower IC50 variability. This is a result that we expect because the drugs whose responses were harder to predict show a wider range of behaviours that are harder to learn for the model.

### 3.2 Learning curves

#### 3.2.1 Learning curves for the intersection of cell lines with RNA-seq and proteomics profiles

**Figure 2.**
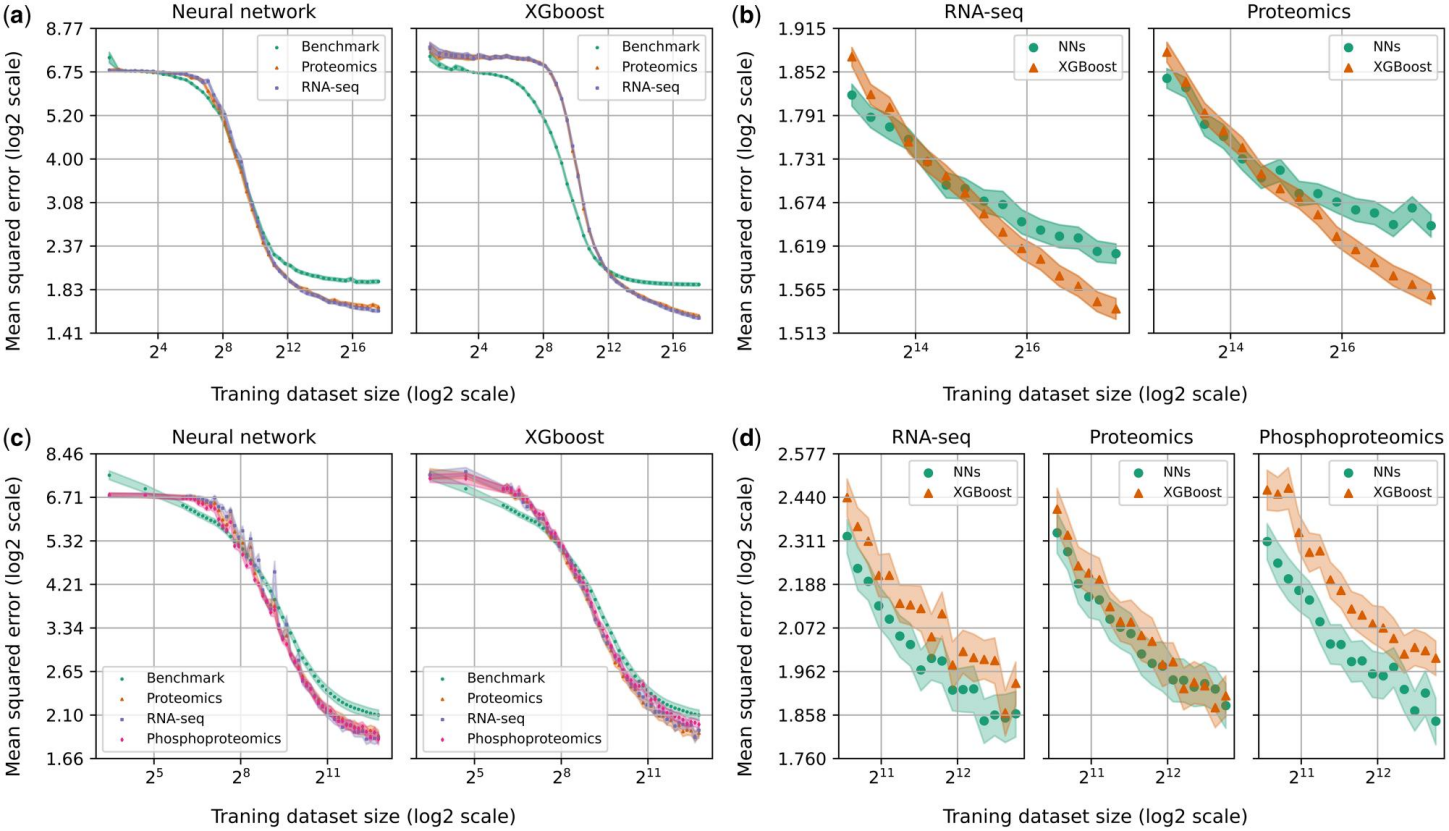
Learning curves for NNs and XGBoost across $CLS_{877}$ and $CLS_{38}$. Shaded regions indicate the standard errors. (a) Learning curves for the benchmark, proteomics, and RNA-seq data in $CLS_{877}$. (b) Comparison between NNs and XGBoost for the larger dataset sizes and $CLS_{877}$. (c) Learning curves for: the benchmark, RNA-seq, proteomics, and phosphoproteomics data in $CLS_{38}$ and the benchmark. (d) Comparison between NNs and XGBoost for the larger dataset sizes and $CLS_{38}$.

Figure 2a shows the learning curves for proteomics and RNA-seq for NNs and XGBoost. The curves are shown for $CLS_{877}$. The figure shows the small data, power-law and diminishing returns regions identified in the introduction. The plots show that for a given algorithm both omics datasets comfortably outperform the benchmark for larger dataset sizes. The figure also shows that, for smaller dataset sizes, NNs outperform XGBoost and *vice versa* for the maximum dataset size. The comparison between XGBoost and NNs is shown in more detail in Fig. 2b. This figure clearly shows that XGBoost outperforms NNs for the larger dataset sizes, for both types of omics data. This figure also shows that XGBoost starts to outperform NNs at a dataset size of $∼2^{15}$ for both RNA-seq and proteomics. Furthermore, the trajectory of the curves, for both data types, shows that the performance of the NNs starts to plateau more quickly than XGBoost. This suggests that with more data XGBoost will increase its performance advantage over NNs.

The learning curves in this report show that for cancer-blind evaluation both algorithms and data types reach the region of diminishing returns. In this region, extra data will increase the performance of the models but not as significantly as the performance improvements seen at smaller dataset sizes. This is in contrast to a study that used mixed-set evaluation and RNA-seq data, which showed that these learning curves, were still in the power-law region (Partin *et al.* 2021). Therefore, while the learning curves plotted here show that more data will improve the model’s performance, these performance improvements are expected to be less than when using mixed-set evaluation. Furthermore, because these models are not in the power-law region, reliable predictions about how much of a performance improvement would be seen with additional data cannot be made.

The learning curves also show a similar performance improvement trajectory is expected for both data types. Furthermore, as shown in Table 1 RNA-seq only slightly outperforms proteomics. This has important consequences in a stratified medicine context because factors, such as speed and cost of data collecting, may be the most important factors when deciding between the data types when current performance and expected performance with more data are similar. However, it is important to note that the learning curves also show it is possible for the RNA-seq and proteomics curves to decouple as more data are collected. Consider the NN curves at the point training dataset size =$2^{9}$. At this point, the trajectory of all three curves is the same. Thus, from the curves alone, we cannot predict that the curves will decouple, with the benchmark plateauing quicker than the omics data. Therefore, we cannot rule out the decoupling of the proteomics and RNA-seq curves.

The learning curves also show that the best model is dataset size dependent. At smaller training set sizes NNs outperformed XGBoost and *vice versa* for larger training set sizes. This shows the value of generating learning curves compared with a standard single performance metric that would not capture this difference. The dataset size-dependent performance metric learning curves are particularly useful in a stratified medicine context. As discussed in Section 1, the cancer-blind evaluation, used in this article, is best for simulating how useful a model would be in a stratified medicine problem. For example, consider looking for a model in the literature that performed well on a cancer-blind test set for use in a clinical trial or similar application. It is very unlikely the clinical trial would have the same dataset size as the one used in a given study of DRP. Instead, it would be more appropriate to use learning curves, such as the ones produced in this report, to find the best-performing model for the size of the trial. The above is also an argument for the general use of learning curves when evaluating a model using a cancer-blind set.

#### 3.2.2 Learning curves for the intersection of cell lines with RNA-seq, phosphoproteomics, and phosphoproteomics profiles

The graphs in Fig. 2c show the learning curves for phosphoproteomics, proteomics, and RNA-seq for NNs and XGBoost. The curves are shown for $CLS_{38}$. Note that the RNA-seq and proteomics curves are not just arcs of the curves in Fig. 2a. They are new curves constructed only with cell lines that are in $CLS_{38}$. These plots again show the three learning regions identified in the introduction. The plots also show that for the larger dataset sizes, all of the omics data outperform the benchmark for both algorithms. The trajectory of all of the curves suggests that more data would improve the algorithm’s performance and for proteomics and RNA-seq data, we know this to be true from the previous learning curves.

The comparison between XGBoost and NNs is shown in more detail in Fig. 2d for the three different types of omics data in $CLS_{38}$. The performance between models is largely similar for all data types. However, NNs do generally perform slightly better than XGBoost. This agrees with the results from $CLS_{877}$, whereas Fig. 2a and b shows for smaller dataset sizes NNs outperform XGBoost. However, it is expected as more data are collected XGBoost will start to outperform NNs as seen in Fig. 2b for the same reasons discussed above.

Currently, there is not a phosphoproteomics dataset that rivals the size of the proteomics or RNA-seq datasets. The one used here has 20 times fewer cell lines. The learning curves plotted here give a way to project the performance of phosphoproteomics forward. Therefore, they can be used for a more informed cost-benefit analysis to determine if more data should be collected.

Consider Table 1, which shows that phosphoproteomics has the lowest MSE using the overlapping cell lines. Furthermore, the performance trajectory of all three omics data types are similar, as shown by learning curves in Fig. 2c. This suggests that phosphoproteomics may perform similarly or slightly better than RNA-seq using datasets of the same size. This would equate to a reduction in MSE of ∼15% relative to the dataset used here.

However, it is also possible the phosphoproteomics learning curve could decouple from the other omics data as happened for the benchmark in Fig. 2a. This decoupling could unfold such that phosphoproteomics has better or worse performance than the RNA-seq and proteomics data.

## 4 Conclusion

The comparison of RNA-seq, proteomics, and phosphoproteomics using the 38 intersecting cell lines ($CLS_{38}$) showed that phosphoproteomics had the lowest MSE of the three omics types. This makes it the best-performing modality for DRP, in terms of MSE. However, RNA-seq performed similarly and had the lowest average ranking across the 30 different train test splits used. The learning curves showed that the performance trajectory of all three omics data types were similar, with the performance of all omics types predicted to increase as more data are collected. Furthermore, for the comparison of RNA-seq and proteomics, it was shown that moving from $CLS_{38}$ to $CLS_{877}$ (the 877 intersecting cell lines with proteomics and RNA-seq profiles) increased the performance advantage of RNA-seq over proteomics. These results suggest that the performance gap between phosphoproteomics and RNA-seq might widen using a dataset of ∼1,000 cell lines. Collecting such a phosphoproteomics dataset would, therefore, be an important future direction for determining the suitability of phosphoproteomics for stratified medicine.

Despite the differences in performance of the three omics modalities, they all performed well—the largest difference in performance was 2% for a given algorithm. Therefore, for different applications of DRP, factors other than predictive performance may be more appropriate in deciding the best modality.

We have also shown that for sufficiently large dataset sizes in $CLS_{877}$, XGBoost outperforms the NN architecture we used. Furthermore, the trajectory of the learning curves suggested that XGBoost would increase its performance advantage over NNs as more data are collected. This suggests that XGBoost should more commonly be used for DRP. The fact different models perform better on different dataset sizes also demonstrates the value of learning curves.

Cell lines are an invaluable resource for cancer research and they are the most common data type used in training DRP models (Baptista *et al.* 2021, Chen and Zhang 2021, Partin *et al.* 2023). Therefore, due to data availability, we restricted our analysis to cell lines. However, using cell lines does have limitations because they do not capture the full complexities of clinical samples. Therefore, applying our work to clinical data or data that more closely represent human tumours, such as patient-derived xenografts is an important future direction.

## Supplementary Material

vbad190_Supplementary_Data

## Data Availability

Code is available at: https://github.com/Nik-BB/Learning-curves-for-DRP.
