# Supplementary material for: Comparison of multiple modalities for drug response prediction with learning curves using neural networks and XGBoost
Source: Bioinform Adv. 2023 Dec 23;4(1):vbad190. doi: 10.1093/bioadv/vbad190 (PMC10812874; doi:10.1093/bioadv/vbad190)
Supplement: vbad190_Supplementary_Data [file vbad190_supplementary_data.pdf]

Supplementary material for Comparison of multiple modalities for DRP paper

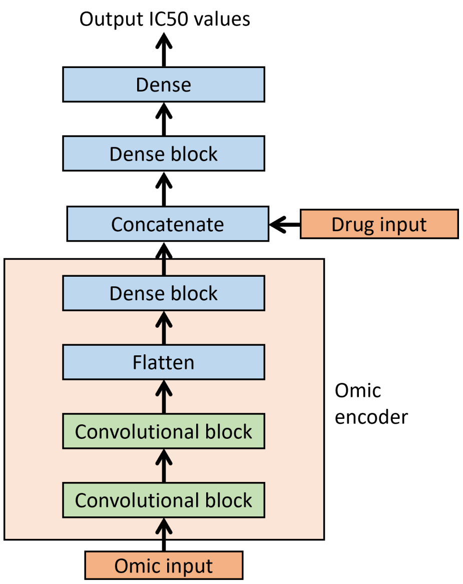

Fig. 1. NN architecture used in this report. The convolutional blocks contained a $1D$ convolutional layer followed by a max pooling layer. The dense blocks contained two dense layers. The ReLU activation function was used for the convolutional and dense layers in the dense and convolutional blocks. Linear activation was applied to the final dense layer. The hyperparameters were optimised using Keras tuner O'Malley et al. (2019)

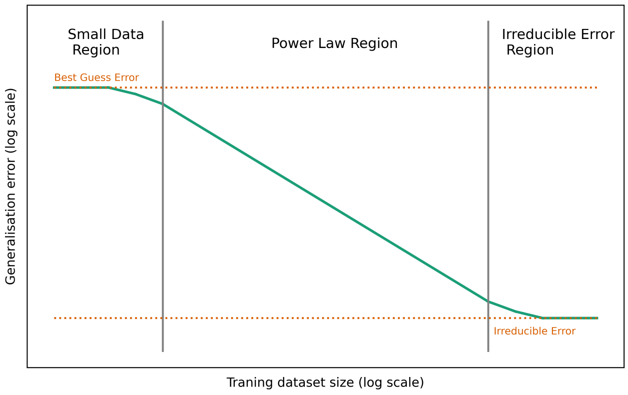

Fig. 2. Sketch of the typical shape of a learning curve adapted from Hestness et al. (2017). The sketch is split into three parts the small data, power-law and irreducible error regions.

Learning curve generation

This section fully details the dataset-splitting method used in order to create the learning curves. The method we used to create the learning curves is similar to the method used in Partin et al. (2021). Consider a dataset D that contains j different cell lines CLs. The CLs in D were split into three sets a: training (T) validation (V) and testing (E) set, according to the fractions(f_T, f_V, f_E) relative to j. For CLS_{877} (the cell line set containing the cell lines that intersect the proteomics and RNA-seq datasets) $f_T = 0.8, f_V = 0.1$ and $f_E = 0.1$. For CLS_{38} (the set that contains cell lines that intersect all three omics datasets) $f_T = 0.6, f_V = 0.2$ and $f_E = 0.2$. The larger testing and validation set sizes were used for CLS_{38} because it was much smaller than CLS_{877}. For example, using $f_E = 0.1$ for CLS_{38} would of only given ~ 4 CLs in the validation set. Therefore, more cell lines were required to decrease the variance in the validation set. Once the CLs in D were split into T V and E all the drugs associated with a given cell line, without missing target values, were added to the respective set. After this T V and E contained cell line drug pair input values and the associated truth values. With the key property that no cell line was in more than one set. Therefore, $T = \{\boldsymbol{x_{c,i}}, \boldsymbol{x_{d,i}}, y_i\}_{i=1}^{k_1}$, $V = \{\boldsymbol{x^v_{c,i}}, \boldsymbol{x^v_{d,i}}, y^v_i\}_{i=1}^{k_2}$, $E = \{\boldsymbol{x^e_{c,i}}, \boldsymbol{x^e_{d,i}}, y^e_i\}_{i=1}^{k_3}$. Such that $\{\boldsymbol{x_{c,i}}\}_{i=1}^{k_1} \cap \{\boldsymbol{x^v_{c,i}}\}_{i=1}^{k_2} = \varnothing$, $\{\boldsymbol{x_{c,i}}\}_{i=1}^{k_1} \cap \{\boldsymbol{x^e_{c,i}}\}_{i=1}^{k_3} = \varnothing$ and $\{\boldsymbol{x^e_{c,i}}\}_{i=1}^{k_3} \cap \{\boldsymbol{x^v_{c,i}}\}_{i=1}^{k_2} = \varnothing$. Where k_1, k_2 and k_3 are the total size (number of drug cell line pairs) of T V and E respectively and the superscripts v and e are just notation to differentiate between the sets.

To create an individual learning curve, T was split into K different sized subsets of size n, $\{T_{n_k}\}_{k=1}^{K}$. Following the logic from Partin et al. (2021), the subsets were sampled from T to mirror how a researcher would exploit gaining access to additional data given an existing dataset. Specifically, the researcher would add this additional data to their existing dataset. Mathematically, given a training data set, T_{n_k}, $T_{n_{k+1}}$ was constructed by adding additional samples to T_{n_k} from T. Therefore, $T_{n_{k-1}} \subset T_{n_k}$ where $n_{k-1} < n_k$.

For a given learning algorithm, at each T_{n_k} a hyperparameter optimised model was selected. The hyperparameters were optimised with a 15 trial random search and early stopping. The validation set V was used for early stopping and to find the optimal hyperparameters. Note that the same validation set was used for all training dataset sizes. Finally, the performance of the model was measured on the test set, E. This gave the generalisation error of the model for a given training set size, s_{n_k}.

The above process was repeated 30 times where D was first shuffled according to a random seed before splitting. The learning curve was found by taking the mean of s_{n_k} across the repeats.

In total $30 \times 15 \times K$ models were created for each learning curve. For CLS_{877} $K = 45$ and for CLS_{38} $K = 52$. The above process was followed to create all 14 learning curves (LCs) in this report. A LC was created for both learning algorithms, DL and XGBoost and data types in CLS_{877}, RNA-seq and proteomics. For CLS_{38} LCs were created for RNA-seq proteomics and phosphoproteomics, for both learning algorithms. LCs for RNA-seq and proteomics had to be created for each CLS to make a fair comparison with the phosphoproteomics data.

Two benchmark learning curves were also created, one for each CLS. It is important to note that for a given CLS and repeat the cell drug pairs used for training validation and

	EMDR	Average ranking	PCA	Average ranking	all-features	Average ranking
Phosphoproteomics	1.87±0.05	2.0	1.99±0.06	1.93	1.88±0.06	1.93
RNA-seq	1.86±0.06	1.83	2.01±0.06	2.07	1.87±0.05	1.93
Proteomics	1.90±0.06	2.17	2.01±0.05	2.0	1.87±0.05	2.13

Table 1. Mean squared error (MSE) and average ranking across the 30 test train split evaluated using the test sets and trained using the full training sets for CLS_{38}. These results are shown for three different feature selection techniques. Neural networks were used for each feature selection technique. The same network architecture and hyperparameter tuning process described in the methods section were used for each feature selection technique, apart from principal component analysis (PCA) that used a kernel size of 4 in the first convolution layer and 2 in the second. Empirical markers of drug responses EMDRs feature selection used the EMDRs identified by Gerdes et al. (2021) for a given omics type. For the all-features method, all of the features in the respective omics types were used.

testing were the same across all data types. All models, apart from the benchmarks, were trained using the GPUs in the Andrena cluster in Queen Mary's Apocrita HPC facility.

Hyperparameter search space

The hyperparameter search space for the neural networks used in this report was as follows:

- Filters in $1D$ convolution layers: (8, 32) step size 8
- Neurons in first dense block: (32, 258) step size 32
- Neurons in second dense block: (32, 258) step size 32
- Learning rate: 1×10^{-4} or 1×10^{-5} for CLS_{38}
- Learning rate: 1×10^{-4} or 1×10^{-3} or 1×10^{-2} for CLS_{877}

Note that for a given trail the filters in both convolution layers and the neurons within a dense block were the same. However, there could be a different number of neurons in the first and second dense blocks. These hyperparameters were optimised using a random search implemented using the Keras tuner package O'Malley et al. (2019).

The fixed hyperparameters were as follows:

- Kernel size = 16 in the fist convolution layer.
- Kernel size = 8 in the second convolution layer.
- Pool size = 2 in the max pooling layers.
- Neurons = 1 in the final dense layer.

The hyperparameter search space for XGBoost models used in this report was as follows:

- Max depth: $\{3, 5, 6, 10, 15, 20\}$
- Learning Rate: $\{0.1, 0.2, 0.3\}$
- Subsample: $(0.7, 1.0)$ step size 0.1
- Columns sample by tree: $(0.4, 1.0)$ step size 0.2
- Alpha regularisation: $\{0, 0.1, 1, 10\}$
- Lambda regularisation: $\{0, 0.1, 1, 10\}$

The maximum number of estimators was set to $25,000$ with early stopping used and set to trigger if no improvement is seen on the validation set within 500 boosting rounds. The reminding hyperparameters were left at their default values as specified by the XGBoost documentation.

Error analysis

Figure 3 shows the distribution of errors for each model and data type combination for CLS_{877}. The models were trained using the maximum training dataset size. The errors are shown for all 30 test train splits. Figure 4 shows the error distribution for CLS_{38}. All histogram in both plots follows an approximately normal distribution centred around 0. Therefore, the models are not making biased predictions above or below the target values. Furthermore, because distributions are

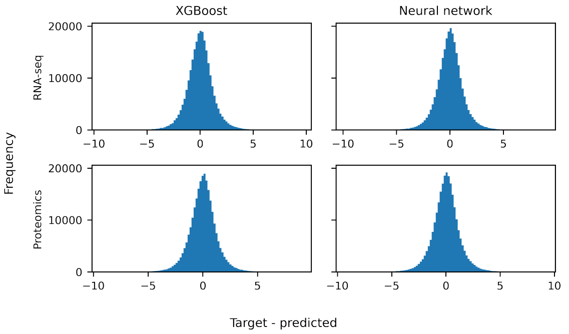

Fig. 3. Plot to show the distribution of the errors on the test set for neural networks and XGBoost for Proteomics and RNA-seq. The histograms give the difference in target and predicted values for each model and data type combination using CLS_{877}. The plots show this for the maximum training dataset size. Furthermore, the error of all test train splits is shown.

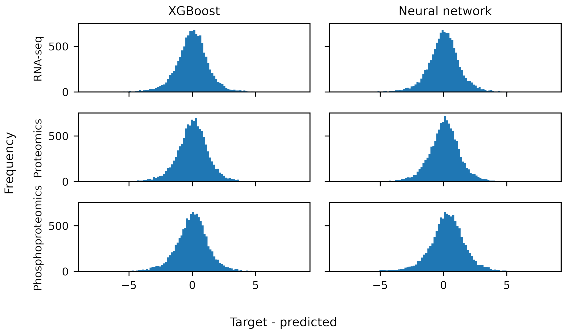

Fig. 4. Plot to show the distribution of the errors on the test set for neural networks and XGBoost for Proteomics, Phosphoproteomics and RNA-seq. The histograms give the difference in target and predicted values for each model and data type combination using CLS_{38}. The plots show this for the maximum training dataset size. Furthermore, the error of all test train splits is shown.

centred around 0 this shows that the predictions are generally accurate.

Per drug analysis

We looked in detail at the 10 drugs whose response neural networks struggled to predict the most and whose responses were predicted most accurately. Figure 5 shows a boxplot of the IC50 values of these drugs that are shared between all three omics types. This suggests that the models perform better for drugs with lower variability in their IC50 values. This is further confirmed by figure 6, which shows that the worst

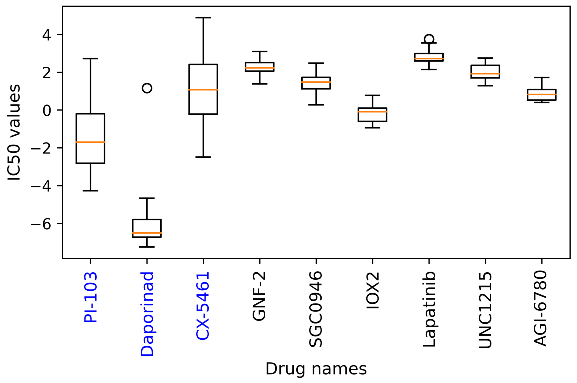

Fig. 5. Boxplot of the IC50 values for the drugs neural network performed the best and worst on. The first three drugs in blue are the drugs whose response the models struggled to predict the most. The drugs whose responses were predicted most accurately are the six other drugs in black. Only the drugs the model performed the best or worst on across all three omics types are shown. The figure shows that the drugs the model performed the best on have a larger spread of IC50 values than the drugs the model performed the worst on.

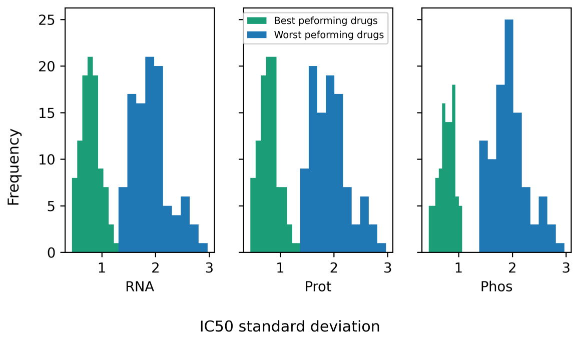

Fig. 6. Distributions of the standard deviations of the IC50 values for the 100 drugs the model performed the best and worst on for neural networks. The distributions are shown separately for RNA Proteomics and Phosphoproteomics omics types. The distributions show that the drugs the model performed the worst on have a larger standard deviation than the drugs the model performed the best on.

performing 100 drugs have smaller standard deviations than the best performing 100 drugs.

References

H. Gerdes, P. Casado, A. Dokal, M. Hijazi, N. Akhtar, R. Osuntola, V. Rajeeve, J. Fitzgibbon, J. Travers, D. Britton, et al. Drug ranking using machine learning systematically predicts the efficacy of anti-cancer drugs. *Nature communications*, 12(1):1–15, 2021.

J. Hestness, S. Narang, N. Ardalani, G. Diamos, H. Jun, H. Kianinejad, M. Patwary, M. Ali, Y. Yang, and Y. Zhou. Deep learning scaling is predictable, empirically. *arXiv preprint arXiv:1712.00409*, 2017.

T. O'Malley, E. Bursztein, J. Long, F. Chollet, H. Jin, L. Invernizzi, et al. Keras Tuner. `https://github.com/keras-team/keras-tuner`, 2019.

A. Partin, T. Brettin, Y. A. Evrard, Y. Zhu, H. Yoo, F. Xia, S. Jiang, A. Clyde, M. Shukla, M. Fonstein, et al. Learning curves for drug response prediction in cancer cell lines. *BMC bioinformatics*, 22(1):1–18, 2021.
